# New Instruments

**Published:** 1878-12

**Authors:** 


### New Instruments.

Rapid and Forcible Dilatation of Cervical Canal for Removal of Offending Bodies. With Descriptions of Instruments for the Purpose. By H. T. Hanks, M. D., New York.—Two years ago, a patient of Dr. W. H Hall, who had previously suffered from epilepsy, became pregnant. She had given birth to two healthy children before, and had carried them to full term without serious inconvenience to herself. At this time, however, the epileptic seizures became exceedingly frequent and alarmingly severe—so much so, indeed, that Dr. Hall and myself both decided that, since medicines had apparently no control in averting the attacks or mitigating their severity, the only safe and judicious course to pursue would be to cause the expulsion of the two months fœtus. The patient lived a few miles out of the city, and it was the wish of the family that Dr. Hall and myself should take full charge of the case without calling in the local physician. Under these circumstances, it became a question with me how to safely and thoroughly accomplish this end and make but one visit myself, and cause as little journeying as possible for Dr. Hall, and not oblige any physician to call until the following day.

I finally decided to rapidly and forcibly dilate the canal, give a full dose of ergot hypodermically, and then remove the ovum by means of the placenta forceps or loop of wire.

Knowing how little dependence can be placed upon Molesworth's, Barnes', or the soft rubber air dilators, when they have been once in use and laid aside for a few months, and knowing from experience how well the healthy unimpregnated cervix uteri will bear rapid dilatation up to No. 24, American scale of bougies, without laceration, I decided to dilate in this case in the most rapid manner possible without injury to the parts involved.

We therefore anæsthetized the patient, introduced a speculum, seized the anterior lip, and in regular order introduced the common hard rubber dilators (see *Medical Record*, Sept. 25, 1875, page 655), from No. 16 up to No. 22, then changed to the smaller sized rectal bougie, and from this to the next larger, and so on up to No. 10. At this time a full dose of fluid extract of ergot was given hypodermically, after which the placenta forceps were introduced and the ovum was quickly removed. The whole operation did not last forty-five minutes.

The patient made a quick and in every way satisfactory recovery.

Since this time I have witnessed the bursting of several of Molesworth's and Barnes' dilators, and I thought much of hard rubber dilators, to take the place of these perishable soft rubber ones. I have therefore had the Messrs. Stohlmann, Pfarre & Co., of this city, manufacture for me a set of *ten* dilators, attached to a double-curved handle.

Each dilator is hollow and more or less ovoid according to its size. The length varies from 6½ to 8½ ctm.; the diameter from 12 m. m. to 5 cm., in proportion to the length. The larger end of each is made to fit either end of the screw-handle rod, which is curved in opposite directions at each extremity, flattened on one side, for security of grasp, and about 13 ctm. in length.

In using these dilators, it is absolutely necessary to have a strong tenaculum forceps in order to firmly fix the cervix. The Messrs. Stohlmann, Pfarre & Co. have made for me a very convenient tenaculum forceps, which will be found to fully meet the requirements of each case, and has never yet been known to tear the cervix.

With this instrument I seize the anterior lip—one blade inside, the other opposite—fully 1 m. m. from the external os, and firmly fix the cervix, either with or without the use of a speculum, according as it is expected to have much or little trouble in removing the contents of the uterus. I then gently but firmly force the smallest dilator into and through the external os, and entirely enter the cervical canal. This end is then withdrawn, and the opposite and next larger dilator is in like manner pushed into the canal. When this end is tightly held within the canal, the smallest dilator, which is now grasped by the hand, is unscrewed and a larger one is screwed in its place. Then the ends are again reversed, and the same course is pursued until the canal is of sufficient size to allow the quick and safe removal of the offending body.

I have used this set of dilators with entire satisfaction in four cases. One patient I saw in consultation with Dr. J. R. Cypert, one in consultation with Dr. D. C. Comstock, and two in my own private practice, besides the one patient in which I used the rectal bougies as before mentioned, and in which the operation consisted in dilating in a similar manner.

The patient of Dr. Cypert had carried a dead three months fœtus for several weeks when the doctor first saw her. She had lost much blood. He applied a tampon after introducing a small sponge-tent. On the following day I saw the lady with him, and on giving ether we easily dilated with these instruments here described, and in about thirty minutes removed the bones and some other portions of the dead fœtus.

The patient made a good recovery.

Dr. Comstock's patient was six weeks pregnant when attacked with uterine hæmorrhage. He applied a tampon and gave anodynes. She had but little subsequent hæmorrhage, but suffered from slight backache pains, and an offensive, dark-colored discharge from the vagina. On my visit I found the external os the size of the little finger, while the internal os was well dilated, and by making firm pressure a loose, fleshy body, the size of a pigeon's egg, could be felt near the dilated internal os. The patient was in good physical condition, and we decided to deliver the ovum without giving ether or using a speculum. The anterior lip was seized with but little difficulty, and the four smaller sizes of the dilators in regular order quickly introduced, and in exactly sixteen minutes from the time I took my place at her side the ovum was removed. It was found to have been filled with a large clot of blood which had completely obliterated the embryo.

My own cases were much like this last. In both, the question of relieving the patient from the danger of hæmorrhage and hours of suffering, together with the saving of much time for myself, came up, and was decided for me by the patients, who were eager to have the anxiety and suffering ended.

One patient had lost "great quantities" of blood before my arrival. The instruments were used in both cases without ether, with success, and both patients did well.

In conclusion, I can recommend the dilators, and should advise their use, where the cervix is healthy, and it is necessary to remove:

1. A retained small or large piece of placenta.
2. A fibrous polypus or tumor.
3. An aborted ovum or dead fœtus.

Even in cases where the child is viable, and it is found necessary (as in placenta prævia) to dilate rapidly, I am certain these instruments would accomplish quickly and safely, in judicious hands, what no other dilators have ever done.

For sale by Messrs. Sharp & Smith.

Description of a Flexible Metallic Uterine Sound and a Flexible Metallic Probe.—By Edward W. Jenks, M. D., Professor of Medical and Surgical Diseases of Women, and Obstetrics in Detroit Medical College.—Every one who has had occasion to explore the uterus where there were any adventitious growths within it, or where from any cause there was an abnormality in either the direction or length of its cavity, can well appreciate the value at times of a flexible uterine sound as a diagnostic aid. Silver, copper, lead, whalebone, rubber, and varions other substances have been used as materials of which were manufactured uterine sounds that could be easily bent so as to fit into the sinuostities of the uterine cavity. In flexures of the uterus a sound that is capable of being adapted to the angular-shaped cavity is sometimes an important desideratum. It is needless to write at any length of the value of flexible uterine sounds or the variety of conditions where they can be advantageously used in diagnosis.

I venture to describe very briefly a flexible uterine sound and a probe, both of which I have used with so much satisfaction for several months that I believe they possess merits which will commend them to the profession.

The sound is 32 ctm. in length, including the hard rubber handle; the material of which the sound is composed is white metal; the entire instrument, including the handle, is hollow.

At the uterine end only, for a distance of 9½ ctm., is there flexibility. The flexibility is effected by a thin strip of metal wound in a spiral manner, and yet when thus wound the shape is the same as the ordinary uterine sound.

There is also upon the sound a scale of inches and fraction, with a slide and screw, by which means the operator can tell while it is within the uterus the depth to which it has entered.

This instrument can also be used as a medium for making liquid topical applications to the uterine cavity. At one portion of the handle, the nozzle of an ordinary hypodermic syringe can be inserted and fluid conveyed through the length of the sound until the spiral is reached, when it escapes through the interstices in a similar manner as from a uterine drop syringe. The instrument in a similar manner can be cleansed and then all danger of conveying infection from one patient to another avoided.

The probe is composed of the same material as the sound, the principal differences are size and shape. The diameter of the probe is the same as the ordinary silver uterine probe, its length is 25 ctm. including the handle; 11½ ctm. of the uterine end is spiral and flexible. A small bulb on the spiral indicates the normal depth of the uterine canal. This probe like the sound is hollow and like it, a syringe can be used at the handle for making applications to the uterine cavity. I have found this a valuable instrument for use elsewhere than in the uterus. I have used it to explore abscesses about the pelvis and elsewhere, also have made it the medium for carbolizing sinuses and deep-seated abscesses; any fluid inserted in the opening of the handle escapes through the spaces of the spiral, and through a small hole at the other end of the probe. The instrument thus serves other purposes than that of a probe.

For sale by Messrs Sharp & Smith.

---

I EARLY adopted, on antizymotic principles, the administration of from 10 to 30 drops every two, three or four hours, of *sulphurous acid* diluted, in scarlet fever. I treated eleven *severe* cases. The ten treated after its adoption recovered.—*Waterman.*

